# Profiling of phosphorylated metabolites from lung cancer by zeolite loaded Mg–Al–Ce ternary hydroxide (Zeolite@MAC) composite

**DOI:** 10.1016/j.heliyon.2023.e16098

**Published:** 2023-05-06

**Authors:** Rimsha Batool, Batool Fatima, Fahmida Jabeen, Dilshad Hussain, Muhammad Imran, Muhammad Najam-ul-Haq

**Affiliations:** a Department of Biochemistry, Bahauddin Zakariya University, Multan, 60800, Pakistan; b Institute of Chemical Sciences, Bahauddin Zakariya University, Multan, 60800, Pakistan; c HEJ Research Institute of Chemistry, International Center for Chemical and Biological Sciences, University of Karachi, Karachi 75270, Pakistan; d Biochemistry Section, Institute of Chemical Sciences, University of Peshawar, 25120, Pakistan

**Keywords:** Ceria nanoparticles, Zeolite, Phosphorylated biomolecules, Metabolites, Biomarkers

## Abstract

Phosphorylated metabolites are linked to metabolism, and the dysregulation of metabolic reactions brings cancer. Dysregulated levels lead to hyperactivation of glycolytic and mitochondrial oxidative phosphorylation pathways. Abnormal concentrations are the indicators of energy-related disorders. In this work, Zeolite-loaded Mg–Al–Ce hydroxides (Zeolite@MAC) are prepared by co-precipitation and characterized through FTIR, XRD, SEM, BET, AFM, TEM, and DLS. Magnesium–Aluminum–Cerium-Zeolite particles enrich phosphate-containing small molecules. These ternary hydroxides carried out the main adsorption mechanism, which swapped the surface hydroxyl group ligands for phosphate and the inner-sphere complex of CePO_4_. XH_2_O. Cerium plays a significant role in the complexation of phosphate, and adding Mg and Al further helps disperse Ce and increase the surface charge on the adsorbent. ΑTP and AMP are the standard molecules for parameter optimization. Zeolite@MAC enriches phosphorylated metabolites followed by their desorption via UV–vis spectrophotometry. MS profiles for healthy and lung cancer serum samples are obtained for phosphorylated metabolites. Characteristic phosphorylated metabolites have been detected in lung cancer samples with high expression. The role of phosphorylated metabolites is explored for abnormal metabolic pathways in lung cancer. The fabricated material is sensitive, selective, and highly enriched for identifying phosphate-specific biomarkers.

## 1 Introduction

Phosphorylated biomolecules regulate biological processes, and their imbalance leads to diseases like cancer. A common phenomenon in cancer cells is the reprogramming of metabolic pathways. High proliferation of cancer cells requires high energy in the form of ATP and other macromolecules through aerobic glycolysis and oxidative phosphorylation. Mitochondrial oxidative phosphorylation generates ATP in cancer cells and contributes towards metastasis [1,2]. More ATP and oxygen abundance cause the Warburg effect in cancer cells. The extracellular ATP pool in cancer cells is more than normal [3]. The increased cytoplasmic AMP levels activate AMPK (AMP‐activated protein kinase) signaling. This leads to increased energy production and decreased energy consumption [4].

High levels of guanosine triphosphate (GTP) synthesis are associated with cancer produced by inosine monophosphate dehydrogenase-2 (IMPDH2). Overexpression of IMPDH2 elevates IMP or GMP intensity in cancer patients [5]. Among the leading causes of death, lung cancer is the most common. About 85% of lung cancers are non-small cell lung carcinomas [6]. Lung cancer mortality is high because of the difficulty in early diagnosis. In lung cancer, M2-PK (Pyruvate kinase) aids in accumulating phosphometabolites [7]. Tumor cells have the characteristics of metabolic phenotypes, which sustain their higher proliferation rates and resist cell death signals. Metabolic dysregulation helps in cancer cells' adaptation, which can support treatment strategies by targeting tumor metabolic transformations. Hyperactivity of glycolysis in lung cancer metabolism leads to hyper-proliferation of cancer cells producing fatty acids, amino acids, nucleotides, and ATP for cancer cells’ survival. Hexokinase is the enzyme of glucose oxidation leading to more glucose 6-phosphate, ATP, and other metabolites production in cancer cells. Apart from hexokinase, phosphofructokinase, pyruvate kinase, and lactate dehydrogenase are overexpressed in lung cancer. Metabolites involved in these pathways include fructose 6-phosphate, fructose 1,6-bisphosphate, ADP, ATP, phosphoenol pyruvate, and lactate with increased expressions. Phosphoenolpyruvate (PEP) is the precursor for amino acids, nucleic acids, and phospholipids synthesis, which is overexpressed in lung cancer leading to tumor malignancy [8]. Metabolic requirements are doubled in cancer cells which overactivated the metabolic pathways, and subsequently, the amounts of metabolites become higher for cancer cells proliferation and survival. These metabolites can be the therapeutic targets of lung cancer.

Metal oxide affinity chromatography (MOAC) enriches phosphate-containing molecules. Hydrophilic interaction liquid chromatography (HILIC) and ion-exchange chromatography fractionate phosphorylated content before subjecting it to immobilized metal ion affinity chromatography (IMAC) and MOAC strategies. Mass spectrometry records the masses and provides structural information on phospho-content [9]. Multifunctional affinity microspheres with TiO_2_ shells were used to purify phosphorylated biomolecules [10]. Phosphate affinity magnetic beads, titanium ions immobilized adsorbent, and silica nanoparticles functionalized with ATP and modified with titanium ions (Ti^4+^-ATP-NPs) were utilized for enriching and purifying phosphorylated metabolites [[11], [12], [13]]. Due to their biocompatibility, it is concluded that metal-based nanomaterials are preferred for phosphate-specific biomolecules from cancer samples. The combination of diverse particles in one material enables the enrichment of phosphorylated peptides in a precise way [14]. Nanoparticles such as graphene oxide can enrich on-target phosphorylated biomolecules [15]. CeO_2_ has wide applications as a catalyst, biosensor, and ultraviolet absorber [16]. Cerium nanoparticles are coherent to enrich phosphopeptides [17]. Zeolite coatings offer diverse applications, have regular microporosity, and are used for adsorbent, catalytic, or ion exchange processes. These are used as coating material. Zeolite coatings are promising technology that offers a consistent sky-scraping and environment-friendly performance [18]. Zeolites have undergone substantial investigation and are used widely in the industrial and agricultural sectors. Introducing La to zeolite increased its selectivity for phosphate [19]. Lanthanum and Ce are in the same chemical family. They share some characteristics [20]. Zeolite was created and loaded with ternary (hydr)oxides to remove phosphate from wastewater [21]. There are many potential applications for using zeolite-coated Mg–Al layered double hydroxides as the substrate for quickly built infiltration systems to remove Cr(VI) from wastewater [22]. Zeolite microparticles (NaX) showed good corrosion-resistance self-healing abilities when loaded with Ce^3+^ [23].

Phosphorylated compounds enrichment and extraction strategies have been reported in recent literature. A potential selective sorbent for phosphorylated analytes is hydroxyapatite [24]. Another novel sorbent for the selective extraction of these phosphorylated chemicals was prepared by integrating HAP nanopowders into monolithic columns [25]. An aptamer-functionalized porous-polymer-coated solid-phase microextraction fiber was prepared by immobilizing an aptamer with adenosine triphosphate as a ligand on the fiber's surface. With great stability and good reusability, this enriched adenosine triphosphate, adenosine diphosphate, and adenosine monophosphate, respectively [26]. Several urea-formaldehyde (UF) resin monoliths have been used as sorbents for ATP and phosphorylated by-products [27].

Zeolite-loaded Mg–Al–Ce hydroxides were developed to enrich intact phosphometabolites and examined via adsorption isotherms of standard phosphometabolites, i.e., AMP and ATP. The zeolite type is Permutite, a sodium aluminosilicate hydrate (Na_2_O·Al_2_O_3_. xSiO_2_. yH_2_O), synthesized from pottery clay, silica sand, and sodium carbonate. The molar ratio of SiO2/Al2O3 is 2:1 in Zeolites. The main goal was to identify phosphorylated metabolites as biomarkers of lung cancer. Zeolited-based materials are highly specific to capture phosphate group containing analytes; therefore, they enrich phosphorylated metabolites from lung cancer patients as samples for the first time. LC-MS further examines phosphorylated metabolites enriched from complex lung cancer samples. The results depict Zeolite@MAC as the efficient material for phosphometabolites analysis in lung cancer.

## 2 Materials and methods

Chemicals used were zeolite, magnesium nitrate hexahydrate (Mg(NO_3_)_2_·6H_2_O), aluminum nitrate nonahydrate (Al(NO_3_)_3_·9H_2_O), cerium nitrate hexahydrate (Ce(NO3)3·6H2O), ammonia (NH_3_), adenosine monophosphate (AMP), adenosine triphosphate (ATP), hydrochloric acid (HCl) and acetonitrile (ACN). All the chemicals were of analytical grade from Sigma Aldrich and used without further treatment.

### 2.1 Synthesis of Zeolite@MAC

Zeolite-loaded magnesium-Aluminum–Cerium (Zeolite@MAC) was synthesized according to the previous protocol [28] using the co-precipitation method. 1.0005 g magnesium nitrate, 0.7318 g aluminum nitrate, and 1.267 g cerium nitrate were mixed in 200 mL deionized water. 10 g zeolite was added to the above mixture and stirred for 3 h at room temperature. pH was adjusted to 9.5 using an ammonia solution. The composite was stirred for 3 h at ambient temperature. It was kept for 24 h at room temperature. The resultant mixture was centrifuged for 10 min at 6000 rpm in falcon tubes, washed, and oven dried for 24 h at 65 °C. The powder was calcined in a furnace at 400 °C for 2 h. Mg/Al/Ce molar ratio was 2:1:2, and the weight ratio was 1.97 (Mg), 9.20 (Al), and 12.50 (Ce) in MAC@Z.

### 2.2 Enrichment parameters optimization

Zeolite-loaded nanoparticles' binding capacity was optimized by varying the AMP and ATP concentrations. 0.1 M AMP and 0.1 M ATP stock solutions were prepared by dissolving 173.6 mg AMP in 50 mL deionized water and 253 mg ATP in 50 mL deionized water. 1–20 mM AMP and ATP solutions/dilutions were prepared from stock solutions. The ATP and AMP dilutions were analyzed through a UV–vis spectrometer, and their absorbance was denoted as initial concentration (C_o_). The acidic pH was adjusted at 5 with a pH meter. 5 mg Zeolite@MAC was added to each test tube containing different mM AMP and ATP concentrations. The solutions were shaken at 25 °C for 0.5 h at 140 rpm, followed by filtration with Whatman filter paper 1 and analyzed by UV spectrophotometer. Similarly, other parameters were optimized, including pH from 2 to 7, shaking time from 10 to 50 min, and Zeolite@MAC quantities from 5 to 25 mg. The final bound concentrations of ATP and AMP in these millimolar solutions were determined by UV–Vis spectrophotometer. The binding capacity of Zeolite@MAC was calculated by the following equation;

(1) (qe = (C_°_ – Ce)V/m)

Where qe is adsorption capacity, C_°_ is initial concentration, Ce is concentration after adsorption, V is solution volume, and m is mass of adsorbent. The mechanism of interaction between synthesized Zeolite@MAC and phosphometabolites is shown in Figure S5.

### 2.3 Dephosphorylation/Zeolite@MAC regeneration

Zeolite-loaded Mg–Al–Ce hydroxides were regenerated by eluting ATP and AMP. Dried particles from previous experiments were added to 3 mL water. The solutions were adjusted at alkaline pH of 9. These were filtered, and the filtrate was analyzed for the presence of ATP and AMP. The shaking time varied from 10 to 50 min. Optimized pH for ATP and AMP detachment from particles was analyzed by varying pH from 7 to 11. Basic pH enabled the detachment of phosphometabolites from Zeolite@MAC.

### 2.4 LC-MS analysis

Serum samples of lung cancer patients were taken with the prior consent of the Ethical Committee of Nishtar Hospital Multan, Pakistan (Ref. No. M-3 (13)/2018). Samples were collected with prior informed consent of the patients and stored at −20 °C. Proteins were precipitated via acetonitrile precipitation, centrifuged at 14,000 g, and the protein pellet was removed. The liquid samples were filtered to remove any particulate matter. 10 mg Zeolite@MAC was added, and enrichment was performed under the optimized conditions, as discussed above. After elution, the samples were analyzed by LC-ESI-MS.

## 3 Results and discussion

### 3.1 Phosphorylated metabolites interaction with Zeolite@MAC

Zeolite comprises Al_2_O_3_ and SiO_2_ with an isoelectric point (IEP) of ∼4–5. At low pH, the surface chemistry has dominant M-OH^2+^ species for adsorbent for negatively charged compounds. The poor electrokinetic behavior of zeolites resides due to surface Si–OH specie which hinders the selective binding to phosphorylated molecules. Combining Al, Mg, and Ce with zeolite in Zeolite@MAC may improve the enrichment compared to zeolite only due to an increased ratio of metal oxides and reduced Si–OH.

### 3.2 Characterization of Zeolite@MAC

**Fig. 1:**
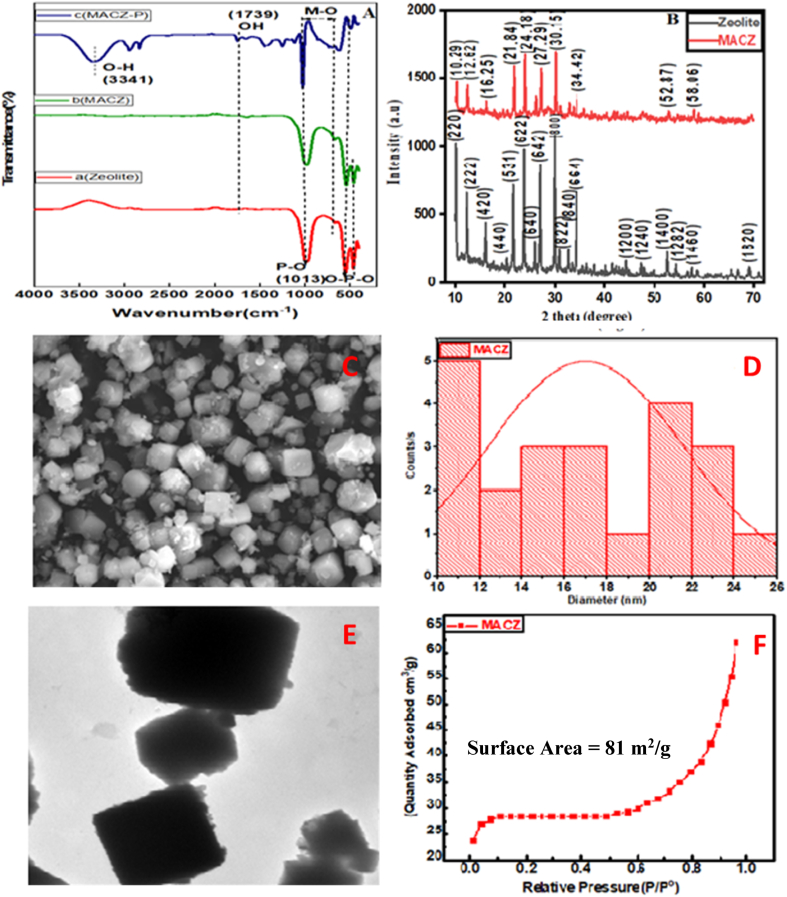
(A) FTIR, (B) XRD, (C) SEM, (D) Particle size distribution, (E) TEM (Zeolite@MAC), and (F) BET.

FTIR analysis confirms the functional groups of zeolite, Zeolite@MAC, and Zeolite@MAC-P (Fig. 1A). Metals are exposed onto the surface of zeolite, and OH groups are in abundance. The surface functionalities assist in phosphate content enrichment. A small peak at 1339 cm^−1^ indicates water's bending vibration and a peak at 3341 cm^−1^ represents OH. 1739 cm^−1^ band shows (NO_3_^−^) ions of salts used in Zeolite@MAC formation. After phosphate adsorption, the band at 3341 cm^−1^ signifies the hydroxyl groups of adsorbent interacting with phosphate groups. *M* − O (M = Si, Al) with *P*–O bond shows a broad peak between 600 and 900 cm^−1^. The inner sphere complex formation is verified by the vibrations of *O*–*P*–O at 454 cm^−1^ and 532 cm^−1^.

The structural crystallinity is examined with X-ray diffraction spectroscopy. A graph is plotted between intensity and 2Ɵ values (Fig. 1B). Diffraction peaks at 10°,12°,16°, 21°, 24°, 27°, 30°, 34°, 52°, and 58° correspond to crystallographic structure of Zeolite@MAC. The values are similar to JCPDS (no.34-0394) database. Raw zeolite exhibits SD card number 01-089-8015. Sharp diffraction peaks of Zeolite@MAC reveal cerium oxide/hydroxide in the main crystallographic structure. Zeolite restricts ternary hydroxides from diffusion into the inner area and is exposed to the nanocomposite's outer surface with hydroxyl groups. XRD peaks at 32°, 34°, 48°, 54°, and 68° indicate the presence of Mg and Al in zeolites structures, whereas Ce is found at 28° and 46°. These elements are present in lower quantities. This is due to the nebulous structure of Zeolite@MAC having higher dispersion of cerium.

The morphology of Zeolite@MAC contributes towards the interactions of metabolites because of enhanced surface area and available surface chemistry. Scanning electron microscopic (SEM) analysis determines the morphological characteristics of Zeolite@MAC. SEM micrographs reveal the regular geometry of particles with hexagonal structures (Fig. 1C). Small particles are attached to the surface of hexagons, indicating Zeolite@MAC formation. The particle size distribution ranges from 150 to 200 nm. Dynamic light scattering (DLS) analysis is also carried out to determine the hydrodynamic diameter of the particles (Fig. 1D). Zeta size results suggest that the diameter of particles is in the range of 200 nm, which is consistent with the SEM measurements.

Transmission electron microscopy (TEM) of as-prepared nanocomposite shows a similar pattern (Fig. 1E). The particles are in polycrystalline form with a uniform hexagonal structure. In addition, TEM images also indicate the homogenous dispersion of Ce, Al, and Mg on the porous zeolite network. TEM image for zeolite is given in supporting material as Figure S1. The morphologies of zeolite and Zeolite@MAC were also analyzed by atomic force microscopy (AFM), where Figures S2A and S2B are the AFM images of zeolite and Zeolite@MAC while Figures S2C and S2D indicate their corresponding particle size distributions. The images and size distribution results also suggest the 15–20 nm range size. Brunauer Emmett Teller (BET) analysis determines the surface area of Zeolite@MAC by N_2_-adsorption isotherm. The measured surface area is 81 m^2^/g, as shown in Fig. 1F. Due to its porous structure, relatively large specific surface area, and improvement of the Ce dispersion, zeolite was demonstrated to be a suitable support material for Ce, all of which increased the efficiency of Ce utilization. The specific surface area of zeolite was reduced in Zeolite@MAC, with the addition of ternary metals, which could be attributed to pore blockage brought on by the precipitation method. This increased the capability of adsorption, compared to zeolite only.

### 3.3 Optimization of enrichment parameters

**Fig. 2:**
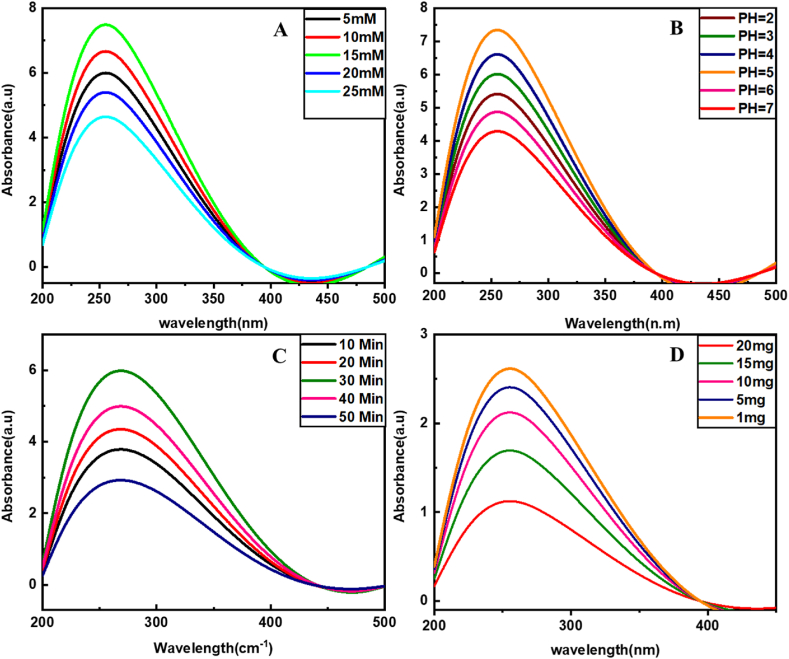
UV–Visible spectra for parameter optimizations, i.e., (A) Concentration of analyte (5–25 mM), (B) Binding pH condition (2–7), (C) Incubation time (10–50 min), and (D) The amount of Zeolite@MAC for adsorption (1–20 mg).

Standard parameters are idealized from the reported literature on phosphate adsorption [29,30]. AMP and ATP are selected as standard phosphorylated metabolites for optimization. The relation between different AMP concentrations and the enrichment capacity of Zeolite@MAC is shown in Fig. 2A. The results reveal 15 mM AMP and 15 mM ATP concentrations. The equilibrium is established above 15 mM of the standard analyte.

The pH condition affects the binding capacity of Zeolite@MAC with analytes. The pH from 2 to 7 is applied, and its effect on binding capacity is measured. The interaction of Zeolite@MAC is feasible in acidic pH as it protonates OH^−^ groups on adsorbent for interaction with phosphorylated biomolecules, including AMP and ATP. In basic conditions, weaker ligand exchange occurs because of deprotonation. ATP shows different peak patterns than AMP because of its more acidic nature. The optimum pH for high adsorption is 5 (Fig. 2B). The suitable incubation conditions improve the adsorption of the target analyte. The incubation uses 15 mM AMP and ATP in Zeolite@MAC with constant mixing for 10–50 min. The UV absorbance and binding capacity results indicate 30 min as the optimum time to achieve maximum loading of phosphorylated analyte (Fig. 2C).

For scrutinizing the efficacy of Zeolite@MAC, different amounts of Zeolite@MAC (1–20 mg) are incubated with 15 mM ATP and AMP at pH 5 for 30 min under continuous stirring (Fig. 2D). The nanocomposite shows a decrease in enrichment with an increase in Zeolite@MAC from 1 mg to 20 mg. These results suggest that 1 mg or <1 mg Zeolite@MAC is the optimum amount required for 15 mM AMP and ATP solutions.

### 3.4 Desorption experiments and regeneration of material

**Fig. 3:**
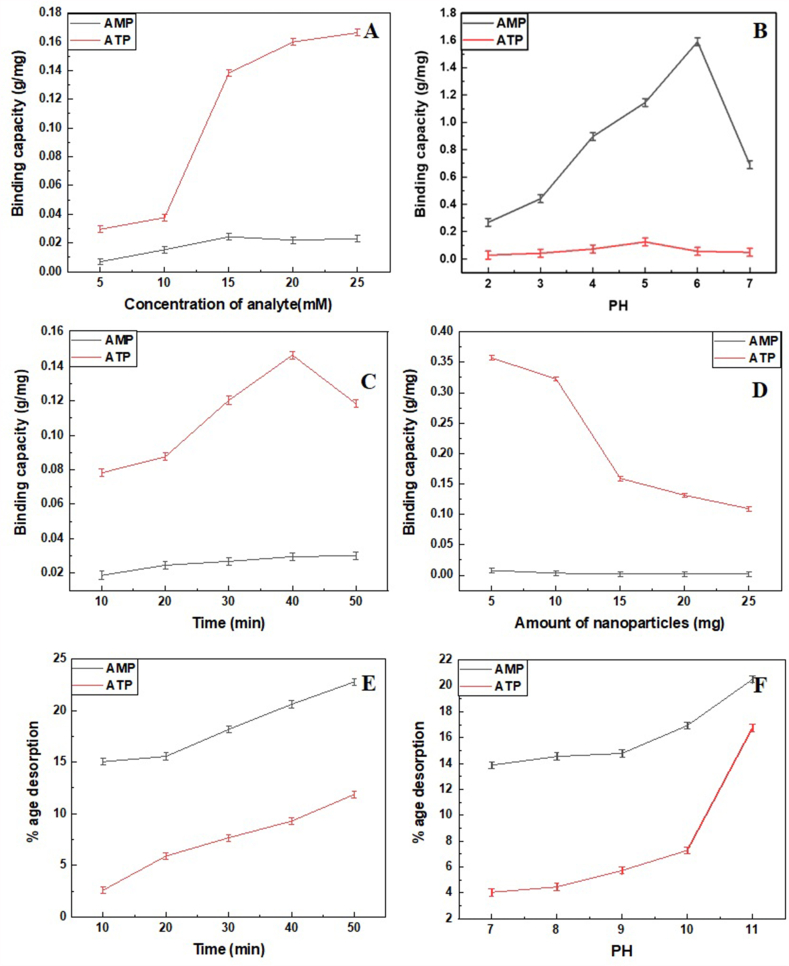
Graphical representation of parameters on the binding capacity of Zeolite@MAC with AMP (black peaks) and ATP (red peaks) at (A) Different concentrations of analytes, (B) pH, (C) Incubation time, (D) Amount of nanocomposite (Zeolite@MAC), (E) Desorption time, and (F) Desorption pH.

The relevant binding capacities of both analytes are also calculated under different conditions to obtain the optimum experimental conditions, and corresponding results in binding capacity are shown in Fig. 3 (A-D). The effects of optimized parameters for dephosphorylating ATP and AMP from particles surface are observed. This ensures the regeneration and reusability of nanoparticles. The amounts of AMP and ATP in the initial filtrate are higher than the amounts after adsorption. However, the amount of analytes in filtrate increased in the final solution after desorption. The following equation calculates the percentage of desorption:

(2) R_D_= (C_d_ x V_d_/ qe x m) x 100

R_D_ is the dephosphorylation efficiency or % desorption, C_d_ is the desorbed phosphometabolite concentration, V_d_ is the volume of solution (L), qe is the adsorbed phosphate quantity calculated at completion of enrichment in mg/g, and m is the mass of Zeolite@MAC (g). The dephosphorylation increases with an increase in shaking time (Fig. 3E). At 10 min, amounts of AMP and ATP in the solution are low, increasing after 20 min. Acidic pH promotes binding, whereas basic pH (7-11) releases bound AMP from the particle surface (optimum pH ∼10), as shown in Fig. 3F.

### 3.5 Adsorption kinetics and isotherms

Phosphometabolite standards enrichment by Zeolite@MAC is studied through adsorption kinetics. Changes in the amount of these phospho-analytes at different times are analyzed. Rapid phosphate enrichment occurs within 30 min, and equilibrium is attained. The homogenously dispersed ternary hydroxides are attributed to the rapid enrichment of ATP and AMP at initial concentration. The electrostatic attraction between the phosphate group and protonated Zeolite@MAC enhances the diffusion of ATP and AMP molecules from the solution towards Zeolite@MAC. Kinetic models, including pseudo-first-order, pseudo-second-order, and intra-particle diffusion, are applied to understand the enrichment mechanism of Zeolite@MAC. The equations are shown below:

(3) Pseudo first order equation: log(q_e_-q_t_) = log q_e_ – k1t/2.303

(4) Pseudo second order equation: t/q_t_ = 1/k_2_ q_e_^2^ + t/q_e_

(5) Intra-particle diffusion equation: q_t_ = k_di_ t½ + C_i_

Where q_e_ and qt are the amounts of phospho-content enriched over a given adsorption period (t) and at equilibrium, k1 (min^−1^), k_2_ [g/(mg^**.**^ min)], and Kdi [g/(mg^**.**^min^0.5^)] are the adsorption rate constants of kinetic models. Ci (mg/g) is related to the thickness of the boundary layer and is a constant of the intra-particle diffusion model. The correlation coefficients show that the pseudo-second-order model represents phosphate adsorption better than the pseudo-first-order model. The rate constants for the pseudo-first-order model are 0.02715 g/(mg. min) for AMP and 0.01261 g/(mg. min) for ATP. The rate constants for pseudo-second-order are 0.3037 g/(mg. min) for AMP and 0.06481 g/(mg. min) for ATP. The rate constants (Kdi) for the intra-particle diffusion model are 326.77814 for AMP and 46.50358 for ATP. It reveals that the synthesized nanoparticles need less time to adsorb phosphate (AMP) at a given concentration. AMP and ATP binding with zeolite@MAC are fast. The ternary hydroxides on porous zeolite enhance the surface adsorption properties for phosphorylated metabolites. The intra-particle diffusion model suggests that phosphorylated metabolites are adsorbed on the surface of particles in the beginning. After saturation of the exterior surface, molecules confiscate in the interior surface of zeolite@MAC due to the intra-particle diffusion. The intra-particle diffusion rate decreases when the interior surface is saturated due to diffusion resistance. The values of K_di_ are higher than K_d2_, indicating that intra-particle diffusion is the rate-controlling process. Adsorption kinetics for zeolite@MAC at initial AMP and ATP concentrations for pseudo-first-order, pseudo-second-order, and intra-particle diffusion kinetics are shown in Figure S3. Adsorption isotherms are also used in the adsorption mechanism. Higher correlation coefficients show that experimental data are well described by the Freundlich equation (R^2^ = 1) and Langmuir equation (R^2^ = 1) for both AMP and ATP (Figure S3 in supporting material). This indicates that multilayers of AMP and ATP are formed on the Zeolite@MAC surface. The efficiency of formulated Z@MAC is compared and shown in Table S1.

### 3.6 Serum analysis by LC-MS

**Fig. 4:**
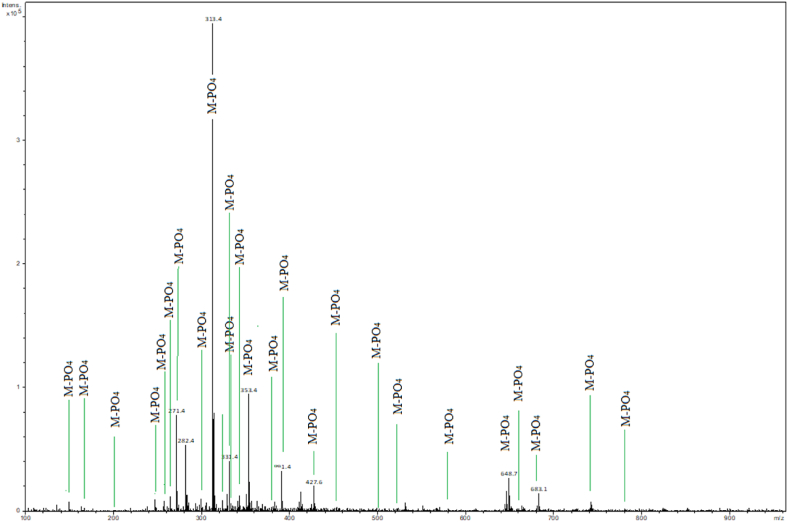
Metabolic profile of lung cancer serum sample corresponding to phosphorylated metabolites enriched by Zeolite@MAC and detected via LC-MS. The identified metabolites are labeled as M-PO_4_.

Metabolic profiling comparisons between cancer and healthy serum samples indicate the overexpression of various metabolites characteristic of abnormal metabolic pathways in cancer. Serum phosphorylated metabolites are enriched using Zeolite@MAC via SPE methodology followed by LC-MS detection. Healthy and lung cancer serum samples are used for metabolic profiling, and 27 phosphorylated metabolites are detected in the lung cancer sample (Fig. 4), whereas only 3 metabolites are detected in the case of a healthy sample (Figure S4). The details of detected phosphorylated metabolites in cancer and healthy samples are provided in Table 1. The extracellular ATP levels rise to hundreds of micromolar due to unprogrammed cancer cell death. High ATP levels in cancer cells also enhance the levels of inflammatory markers [31]. KRas is the GTPase protein that switches between GTP-bound active state to GDP-bound inactive state. GTPase proteins have a role in the cell's growth, proliferation, differentiation, and survival. Oncogenic mutations in KRas proteins lead to lung carcinoma by affecting the GTP-bound and GDP-bound states [32]. The expression of S_1_PR_3_ rises in lung adenocarcinoma, leading to the activation of EGFR [33]. Fructose 1,6-bisphosphate is the intermediate of the pentose phosphate pathway. In cancer, the pentose phosphate pathway is affected, leading to the inactivation and activation of other metabolites [34].

**Table 1:** Details of enriched and detected phosphorylated metabolites by SPE-based Zeolite@MAC followed by LC-MS analysis of healthy and lung cancer serum samples. The monoisotopic masses and metabolic pathway information are provided. Human Metabolome Database (HMDB) is explored to identify enriched metabolites.

Sr. No	Monoisotopic mass	Phosphorylated Metabolite	HMDB ID	Metabolic pathway Involved	Serum samples	
					Healthy	Lung cancer
1	149.1	*O*-Phosphoethanolamine	HMDB0000224	Accumulation in cancer cells during glutamine deprivation	–	*
2	169.1	Phosphoenol pyruvate	HMDB0000263	Inhibited cell growth, proliferation, migration, and invasion and induced robust apoptosis activation	–	*
3	200.1	4-*O*-phosphonic-d-erythrose	HMDB0001321	Involved in the metabolic disorder called the glucose-6-phosphate Dehydrogenase deficiency pathway	–	*
4	244.03	Fucose 1-phosphate	HMDB0001265	Hyperactivity of glycolysis	–	*
5	257.10	Glycerophosphocholine	HMDB0000086	Overexpression in cancer cells	–	*
6	260.02	Glucose 6-phosphate	HMDB0001401	Hyperactivity of glycolysis	*	*
7	265.95	Glyceric acid 1,3-biphosphate	HMDB0001270	Involved in the Warburg effect (aerobic glycolysis), a metabolic shift that is a hallmark of cancer	–	*
8	271.4	Alpha-d-glucuronate 1-phosphate	HMDB0304535		–	*
9	300.1	2-trans,-6-trans-farnesyl monophosphate	HMDB0304088		–	*
10	313.4	5′-Phosphoribosyl-*N*-formylglycinamidine	HMDB0002305	Biochemical intermediate in the formation of purine nucleotides via Inosine-5-monophosphate	*	*
11	323.4	Uridine 5′-monophosphate	HMDB00288	Involved in pyrimidine synthesis down expression in lung cancer	–	*
12	331.4	Deoxyadenosine monophosphate	HMDB0000905	Overexpression in cancer cells	–	*
13	335.4	Fructose 1,6-bisphosphate	HMDB0003973	Hyperactivity of glycolysis	–	*
14	347	Adenosine monophosphate	HMDB0000045	Overexpression	*	*
15	353.9	(2*S*,4*R*,5*S*)-4,5,6-Trihydroxy-2-[hydroxy (phosphonooxy)phosphoryl]oxy-3-oxohexanoic acid	HMDB0260134	Part of the human exposome	–	*
16	379.24	Sphingosine 1-phosphate	HMDB0000277	S1P controls numerous aspects of cell physiology, including cell survival and Mammalian inflammatory responses	–	*
17	427.21	Adenosine diphosphate (ADP)	HMDB0001341	Glucose metabolism	–	*
18	454	phosphoribosylaminoimidazolesuccinocarboxamideSAICAR	HMDB0000797	Act as an oncometabolite Promotes tumor growth and survival	–	*
19	506.9	Adenosine 5′-triphosphate (ATP)	HMDB0000538	Hyperactivity of glycolysis	–	*
20	523	Guanosine triphosphate (GTP)	HMDB0001273	The elevated intensity of Cancer	–	*
21	549.37	LysoPC(20:1 (11Z)/0:0)	HMDB0010391	Over-expressed, potential biomarker, lipid metabolism	–	*
22	578.39	Phosphatidic acid (10:0/17:0)	HMDB0114774	Intermediates in the biosynthesis of triacylglycerols and phospholipids.	–	*
23	648.8	2,3-Bis(palmitoyloxy)propyl dihydrogen phosphate	HMDB0244852	Part of the human exposome	–	*
24	660.47	PA (18:1 (9Z)/15:0)	HMDB0114923	Intermediates in the biosynthesis of triacylglycerols and phospholipids	–	*
25	683.4	Phophatidylethanolamine (32:4)	HMDB0008832	Elevated levels of lung cancer	–	*
26	745.4209	NADPH	HMDB0000221	Hyperactivity of glycolysis	–	*
27	787.17	FADH	HMDB0001197	Hyperactivity of glycolysis	–	*

Metabolic profiling at different cancer stages can help track metabolic abnormalities during tumorigenesis. The detected phosphorylated metabolites represent different metabolic pathways with significant changes due to cancer. In glycolysis, consistent changes result in elevated glucose uptake due to rapid cancer cell mitosis. This results in the overexpression of phosphorylated metabolites such as fucose 1-phosphate (*m*/*z* 244), glucose 6-phosphate (*m*/*z* 260.02), fructose 1,6-bisphosphate (*m*/*z* 335.4), NADPH (*m*/*z* 745.42) and FADH (*m*/*z* 787.17). Glyceric acid 1,3-biphosphate (*m*/*z* 265.9) corresponds to the Warburg effect, which indicates cancer metabolism. Different phosphorylated metabolites like *O*-phosphoethanolamine (*m*/*z* 149.1), lysoPC (*m*/*z* 549.37), phosphatidic acid (*m*/*z* 578.39), PA (*m*/*z* 660.47), and phosphatidylethanolamine (*m*/*z* 683.4) from phospholipid metabolism are detected reflecting the growth of cancer. Phosphoribosylaminoimidazole-succinic-carboxamide (*m*/*z* 454, SAICAR), an oncometabolite, is detected. Its overexpression stimulates pyruvate kinase isoform M2 and promotes cancer cell survival in glucose-limited conditions.

## 4 Conclusion

In this study, the ternary hydroxides of magnesium, aluminum, and cerium coated on zeolite (Zeolite@MAC) are prepared by the co-precipitation method and developed to enrich the phosphorylated metabolites. Adsorption and desorption parameters were done by using a UV–vis spectrophotometer. The average pore size of zeolite is 1.31 nm, which limits metal migration to the inside of the zeolite. Thus, the bulk of ternary (hydr)oxides were dispersed on the zeolite's exterior, making phosphate adsorption easier. Zeolite@MAC nanomaterial is optimized to determine phosphate-specific biomarkers from lung cancer samples. The zeolite adsorbs desired phosphate moieties with high efficiency by increasing the availability of exposed OH groups and providing inner sphere complexation for phosphates. Kinetic study shows rapid and effective AMP and ATP adsorptions on Zeolite@MAC. The phosphometabolites enrichment upon Zeolite@MAC is about 0.024 mg/15 mM (AMP) and 0.14mg/15 mM (ATP). Serum metabolic profiles of lung cancer and healthy samples provide the application of SPE-based Zeolite@MAC on complex biological samples. Because of its higher affinity, lower cost, and improved Ce dispersion, this adsorbent is promising for phosphate enrichment. This is elaborated that the nanomaterial has increased surface area to charge ratio, and its active sites are fully exhibited for the attachment of phosphorylated metabolites. The specificity of these nanoparticles is their usability in the smallest amount (≤0.5 mg) and their reproducibility after desorption. Because of their smaller size and greater surface area, Zeolite@MAC is more competent than other adsorbents. The characteristic enrichment of phosphorylated metabolites via Zeolite@MAC under optimized conditions shows the future perspective of developed nanomaterial in metabolism-specific cancer studies.

## Funding

There is no funding source available for this research.

## Author contribution statement

Rimsha Batool: Performed the experiments; Wrote the paper.

Batool Fatima, Muhammad Najam-ul-Haq: Conceived and designed the experiments.

Fahmida Jabeen, Dilshad Hussain: Analyzed and interpreted the data.

Muhammad Imran: Contributed reagents, materials, analysis tools or data.

## Data availability statement

Data included in article/supplementary material/referenced in article.

## Declaration of competing interest

The authors declare that they have no known competing financial interests or personal relationships that could have appeared to influence the work reported in this paper
