# Supplementary material for: Profiling of phosphorylated metabolites from lung cancer by zeolite loaded Mg–Al–Ce ternary hydroxide (Zeolite@MAC) composite
Source: Heliyon. 2023 May 6;9(5):e16098. doi: 10.1016/j.heliyon.2023.e16098 (PMC10196856; doi:10.1016/j.heliyon.2023.e16098)
Supplement: Multimedia component 1 [file mmc1.docx]

**Supporting Information**

*For*

**Profiling of Phosphorylated Metabolites from Lung cancer by Zeolite loaded Mg-Al-Ce Ternary hydroxide (Zeolite@MAC) Composite**

Rimsha Batool^1^, Batool Fatima^1^*, Fahmida Jabeen^2^, Dilshad Hussain^3^, Muhammad Imran^4^, Muhammad Najam-ul-Haq^2^*

^1^: Department of Biochemistry, Bahauddin Zakariya University, Multan, 60800, Pakistan.

^2^: Institute of Chemical Sciences, Bahauddin Zakariya University, Multan, 60800, Pakistan.

^3^: HEJ Research Institute of Chemistry, International Center for Chemical and Biological Sciences, University of Karachi, Karachi 75270, Pakistan

^4^: Biochemistry Section, Institute of Chemical Sciences, University of Peshawar, 25120, Pakistan.

* Corresponding Author (s)

**Dr. Batool Fatima**

Email: [batoolfatima@bzu.edu.pk](mailto:batoolfatima@bzu.edu.pk)

**Prof. Dr. M. Najam-ul-Haq**

Email: [najamulhaq@bzu.edu.p](mailto:najamulhaq@bzu.edu.p)k

**
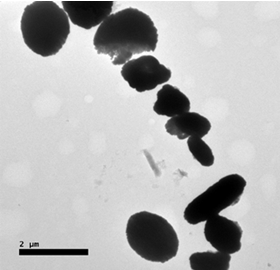
**

**Figure S1.** TEM image of zeolite.


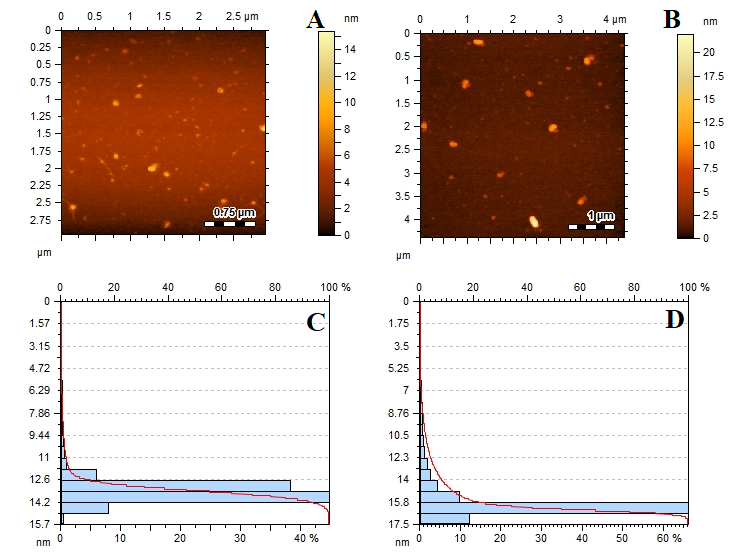


**Figure S2.** AFM images and particle size distribution of zeolite and Zeolite@MAC


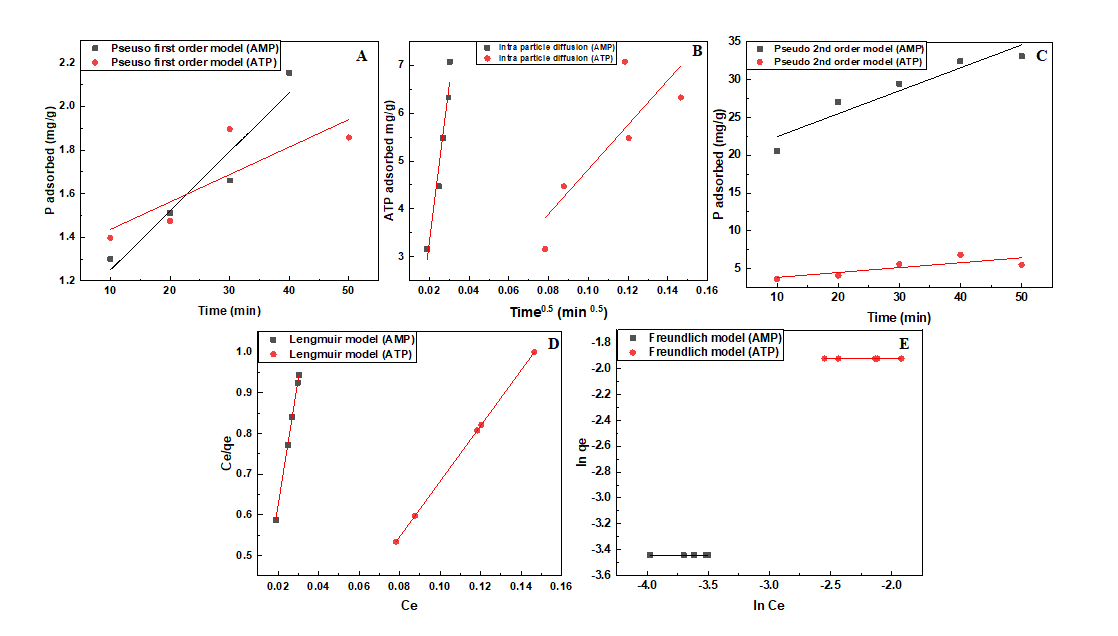


**Figure S3.** Adsorption kinetics for Zeolite@MAC at initial AMP and ATP concentrations, (A) Pseudo first order, and (B) Intra-particle diffusion, (C) Pseudo-second-order, (D) Langmuir model, (E) Freundlich model.


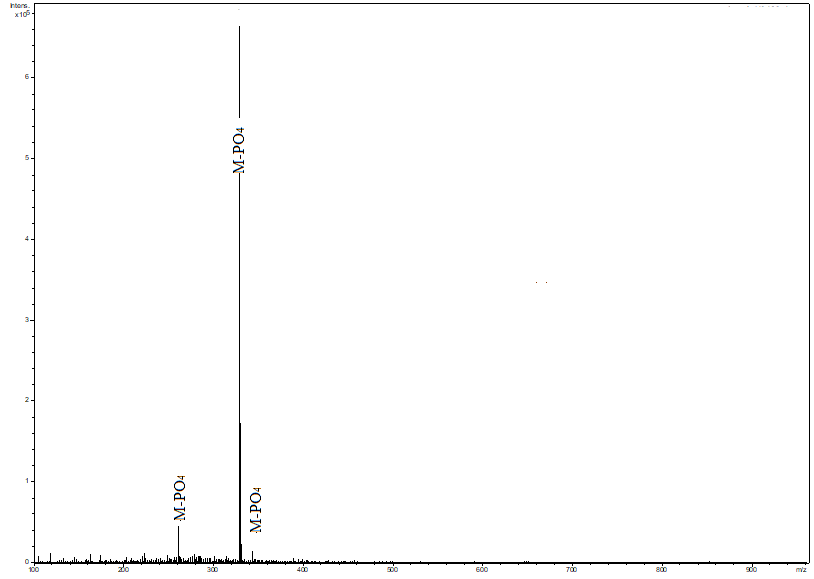


**Figure S4.** Metabolite profile for healthy serum sample representing phosphorylated metabolites enriched by Zeolite@MAC and detected via LCMS. The identified metabolites are labeled as M-PO_4._


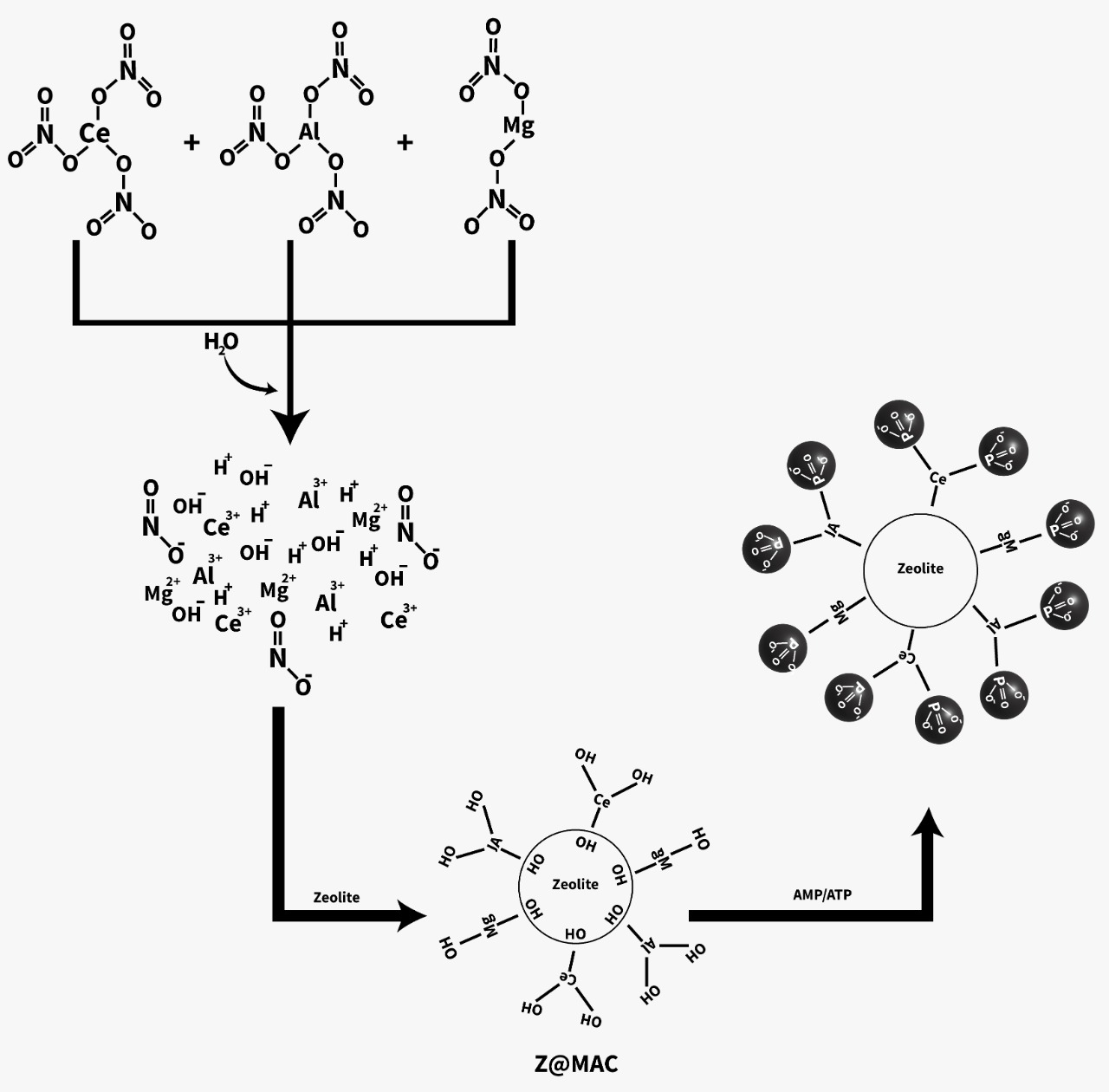
**Figure S5.** Mechanism of phosphate adsorption onto Zeolite@MAC nanoparticles

**Table S1.** The list of already reported nanocomposites for phosphorylated peptides as compared to Zeolite@MAC.

| Nanomaterials | Biological sample or standard | Physical properties | Characterization  techniques | Synthesis method | Sensitivity/  adsorption capacity |
| --- | --- | --- | --- | --- | --- |
| Fe_3_O_4_-MIL-100 (Fe) | β-casein,  α-casein | 168.66 m^2^ g^–1^  1.93 and 3.91 nm | TEM, FTIR,  BET, TGA | Layer by layer method | 60 mg g^–1^  0.5 f mol |
| Zr-OMC | β-casein | 5.6nm,  387m^2^g^−1^ 0.35cm^3^ g^−1^ | SEM, TEM, XPS, EDS, BET, and XRD | Co-assembly (multi-component) with direct carbonization | 1.5 f mol |
| GF-TiO_2_–GO | β-casein | 10–20 nm | SEM, EDX, FTIR | sol–gel method | 1 × 10^−11^ M |
| Fe_3_O_4_@TCPP-DOTA-Tb/Ti | α-casein  BSA | 30 nm | SEM, TEM, EDX, FTIR |  | 94% specificity |
| Fe_3_O_4_@nSiO_2_@mSiO_2_/TiO_2_-Ti^4+^ | nonfat milk  β-casein/  BSA | 179.3 m^2^/g  200 nm | TEM, SEM  EDX, BET | Layer by layer method | 4 pmol |
| DMSNs@PDA-Ti^4+^ | α-casein  nonfat milk  human serum  BSA | 362 m^2^ g^–1^  1.37 cm^3^ g^–1^  ∼150 nm | TEM, SEM,  FTIR, EDX, TGAICP, FEI | Sodium salicylate assisted method | 0.2 fmol/μL  >95% |
| Mg/Al-MMT-La(OH)_3_ |  | 183.49 m^2^/g  0.18 cm3/g  3.7 nm | XPS, EDX, FTIRXRD, SEM,TGA | co-precipitation, ion exchange | 79.33 mg/g |
| CeO_2_ | α‐casein, β‐casein/BSA | 26 nm | TEM, XRD |  | 10 ml |
| CeO2-Fe_2_O_3_,  CeO-SnO2 | β-casein/  BSA |  | FTIR  SEM | co-precipitation method | 1 pmol μL^-1^ |
| Al-LaO_2_, Al-TiO_2_  Al-CeO_2_, Al-ZrO_2_ | β-casein/  BSA | 50-100 nm | SEM  EDX | Co-precipitation | 10 femtomole |
| MC-TiNbNS | β-casein  skim milk  human serum | 60–80 nm  5 nm  128.8 m^3^/g, | SEM, TEM  XRD |  | 2 × 10^–10^ M |
| CeO_2_ | α-casein or β-casein  Human serum  Non-fat milk | 5.0 μm  80 m^2^/g  0.41 cm^3^/g | SEM  XRD  FTIR | staged sol-gel templating protocol | 0.12 μg/ml |
| Zeolite@MAC  (Present Work) | ATP, AMP | 81m^2^/g | XRD, FTIR, SEM, TEM, BET, AFM, | Co-precipitation method | 0.0244mg AMP/15 mmol, 0.14mg/15mmol |
